# Revealing Structure and Localization of Steroid Regioisomers through Predictive Fragmentation Patterns in Mass Spectrometry Imaging

**DOI:** 10.1021/acs.analchem.3c03931

**Published:** 2023-11-17

**Authors:** Varun V. Sharma, Ingela Lanekoff

**Affiliations:** Department of Chemistry − BMC, Uppsala University, 75123 Uppsala, Sweden

## Abstract

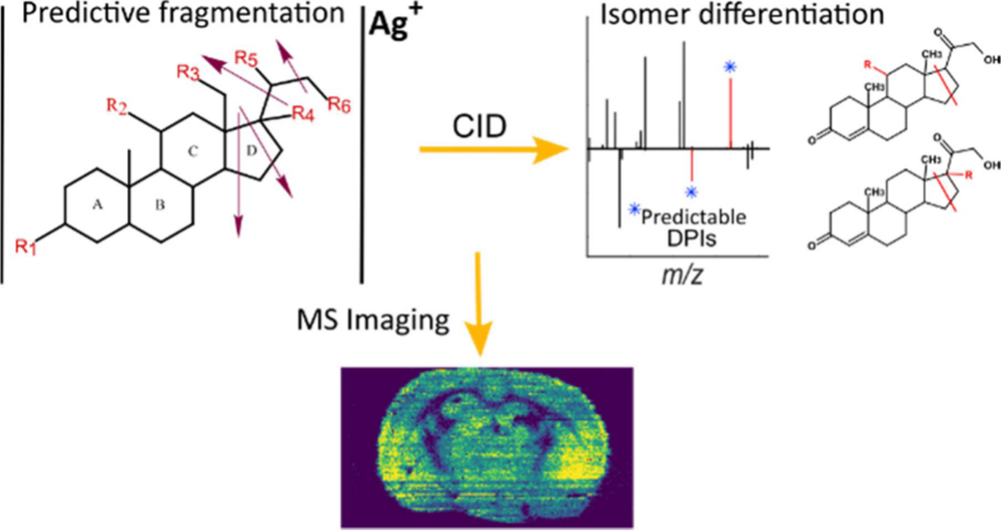

Identifying and mapping steroids in tissues can provide opportunities for biomarker discovery, the interrogation of disease progression, and new therapeutics. Although separation coupled to mass spectrometry (MS) has emerged as a powerful tool for studying steroids, imaging and annotating steroid isomers remains challenging. Herein, we present a new method based on the fragmentation of silver-cationized steroids in tandem MS, which produces distinctive and consistent fragmentation patterns conferring confidence in steroid annotation at the regioisomeric level without using prior derivatization, separation, or instrumental modification. In addition to predicting the structure of the steroid with isomeric specificity, the method is simple, flexible, and inexpensive, suggesting that the wider community will easily adapt to it. We demonstrate the utility of our approach by visualizing steroids and steroid isomer distributions in mouse brain tissue using silver-doped pneumatically assisted nanospray desorption electrospray ionization mass spectrometry imaging.

## Introduction

Steroids are essential signaling molecules that modulate a vast array of physiological processes ranging from neural functions, inflammation, and metabolism to immune responses, electrolyte balance, and reproduction.^1−4^ Consequently, dysregulation of steroids has been linked to several diseases, including neurodegeneration,^3^ cancer,^4^ diabetes,^5^ and cardiovascular diseases,^6^ suggesting their roles as potential biomarkers and therapeutics. The numerous redox reactions that steroids undergo during their biosynthesis produce various isomers with distinct biological activities.^1^ For example, aldosterone is a mineralocorticoid that influences salt and water balance while its biologically inactive structural isomer cortisone converts to the active form cortisol, promoting gluconeogenesis.^7,8^ Steroid functions are intimately linked to their molecular structure and location in tissue,^9^ making it critical to analytically discern isomer distributions to realize their impact on biological function(s) in health and disease.

Mass spectrometry (MS) combined with chromatography is considered the gold standard for studying steroids.^9,10^ Unfortunately, this masks the localization of steroids in tissue. A few steroids have been spatially mapped in tissue with mass spectrometry imaging (MSI) by derivatizing targeted functional groups.^11−15^ For example, tissue derivatization of C=O moiety by Girard T combined with matrix-assisted laser desorption ionization (MALDI) MSI in tandem MS enabled distinct differentiation of two pairs of isomeric species, which pinpointed accumulation of 18-hydroxycortisol in aldo-producing cell clusters in the human adrenal gland.^12,13^ However, mapping steroids without functional group preselection and annotating steroid isomers with MSI would provide further improvements to research on the importance of steroids in biology. Visualizing the distribution is crucial for understanding the role of individual steroid isomers in the chemistry of life.

Despite previous reports on successful strategies for MSI of steroids, challenges include expanding the detectability of steroids beyond those having C=O bonds and enhancing detectability without prior derivatization steps to increase throughput.^16^ Here, we describe a highly sensitive and specific analysis of steroid regioisomers without prior derivatization and limitations to functional groups, such as C=C or C=O/C–OH. This approach combines enhanced detection of steroids through silver (Ag^+^) cationization with the formation of predictable diagnostic product ions (DPIs) by collision-induced dissociation (CID) in tandem MS. Importantly, this enables direct annotation and spatial mapping of steroids using silver-doped pneumatically assisted nanospray desorption electrospray ionization (PA nano-DESI) MSI without any prior derivatization, preseparation, or instrumental modification. Using the developed methodology, we demonstrate imaging of steroids and steroid isomers in thin mouse brain tissue sections.

## Experimental Section

### Materials and Reagents

Solid steroid standards were purchased from Sigma-Aldrich Steinheim, Germany (>98% pure), including pregnenolone, 20α-hydroxyprogesterone, 11α-hydroxyprogesterone, 11β-hydroxyprogesterone, 17-hydroxyprogestrone (17OHP), 21-hydroxyprogestrone, testosterone and dehydroepiandrosterone, cortisol, corticosterone, 21-deoxycortisone, 11-deoxycortisol, estrone, androstanedione, androstanediol, and estradiol and kept at 4 °C. Additional reagents and solvents, including silver nitrate (AgNO3, ≥ 99.8%) and monoisotopic silver nitrate (^107^AgNO_3_, 99.5% purity), were purchased from Trace Science International, Richmond Hill, ON Canada), while acetonitrile [liquid chromatography–mass spectrometry (LC-MS) grade] and methanol (LC-MS grade) were acquired from Merck, Darmstadt, Germany. Water (18.2Ω) was obtained from the Milli-Q purification system (Millipore, Bedford, MA, USA). Fused silica capillaries were purchased from Polymicro Technologies, USA. A high-pressure PEEK tee for the PA nano-DESI with 0.050″ through hole was purchased from Upchurch Scientific Oak Harbor, USA.

### Biological Tissues

Mouse brains were purchased from Creative Biolabs (NY, USA). Tissues were sectioned using a cryomicrotome (Leica Microsystems, Wetzlar, Germany) to a thickness of 12 μm. The sections were thaw-mounted on regular glass slides (Fisher Scientific, Gothenburg, Sweden) and stored at −80 °C prior to the analysis.

### Sample Analysis for Sensitivity and Specificity Studies

Monoisotopic silver was prepared gravimetrically by dissolving in subboiled nitric acid. Excess acid was evaporated, and ^107^AgNO_3_ was weighed. The ^107^AgNO_3_ was dissolved in 20 mL of Milli-Q-water to a concentration of 1100 ppm (m/v); the vial was covered with aluminum foil and stored at 4 °C until use.

All steroid solutions were gravimetrically prepared by dissolving them in methanol to make a 1000 μM stock solution. For fragmentation studies, steroid solutions were diluted with 9:1 methanol/water (v/v) and 10 ppm Ag^+^ solution with natural isotope abundance. For sensitivity comparison between [M + Ag]^+^ and [M + H]^+^ ions, a series of steroid standard solutions ranging from 0 to 0.9 μM were prepared. To promote silver adduction during ESI, steroid standards were diluted in 9:1 methanol/water (v/v) with 10 ppm of Ag^+^ (m/v), while 0.1% formic acid in 9:1 methanol/water (v/v) was used to promote protonation. All solutions were directly infused into Thermo Scientific LTQ Orbitrap Velos. The mass spectra were acquired with a mass resolving power of 100 000 (*m*/Δ*m* at *m*/*z* 400) with a mass window of *m*/*z* 250–500 in positive ion mode. For all fragmentation studies, targeted *m*/*z* was isolated with an isolation width of 1 and fragmented with a normalized collision energy of 20, 30, 40, and 50. Other operating conditions for MS analysis were applied as follows: the source voltage was set to 4.25 kV the inlet capillary temperature was 380 °C; the automated gain control target was 1 × 10^6^ ions; one microscan was used; and the sheath gas flow was 6 arbitrary units.

### PA Nano-DESI MSI

An in-house constructed PA nano-DESI platform was operated as previously described by Duncan et al.^17^ coupled to a Thermo Scientific orbitrap tribrid IQ-X mass spectrometer. Briefly, the PA nano-DESI probe was assembled using a PEEK high-pressure sample tee by inserting fused silica capillaries lengthwise along the tee. Nitrogen gas was fixed into the top of the sample tee by using 0.75 mm Teflon tubing. The angle between the inlet of the MS and the secondary capillary was kept at 150° using a micromanipulator, while the distance between the secondary capillary and the MS inlet was optimized each time the apparatus was assembled. The capillaries were positioned with an 85° angle between the primary and secondary capillaries using micromanipulators. The primary capillary was connected to a syringe pump with a solvent flow of 0.5 μL/min. The nano-DESI solvent for imaging experiments was 9:1 acetonitrile/methanol with 10 ppm of ^107^Ag^+^ (added as ^107^AgNO_3_). Motorized linear stages with *XYZ* configuration controlled by a LabView program with a line space of 75 μm in all imaging experiments resulting in 2x oversampling. MS^1^ mass spectra were acquired in the 370–550 *m*/*z* range with 1 Microscan, 100% AGC target, IT of 200 ms, and a mass resolution of 120,000 (*m*/Δ*m* at *m*/*z* 400). MS^2^ experiments were done by fragmenting mass channels of argenated adducts corresponding to estradiol (*m*/*z* 379.0827), androstanediol (*m*/*z* 399.1453), pregnenolone (*m*/*z* 423.1453), and pregnanolone (*m*/*z* 425.1497) with a mass resolution of 60,000 (*m*/Δ*m* at *m*/*z* 400).

### Data Processing

For imaging, all raw data files were converted to mzXML using Proteowizard MSConvert. Data processing was done using in-house developed i2i software in MATLAB version 2022Rb.^18^ For line scans, mass spectra generation, and calibration curves, signal intensities were extracted from Xcalibur files and transferred to Excel files, and average signal intensities of the specific *m*/*z* over 2 min of the stable signal were plotted using MATLAB version 2022Rb.

## Results and Discussion

### Silver Cationization Increases Sensitivity

**Figure 1:**
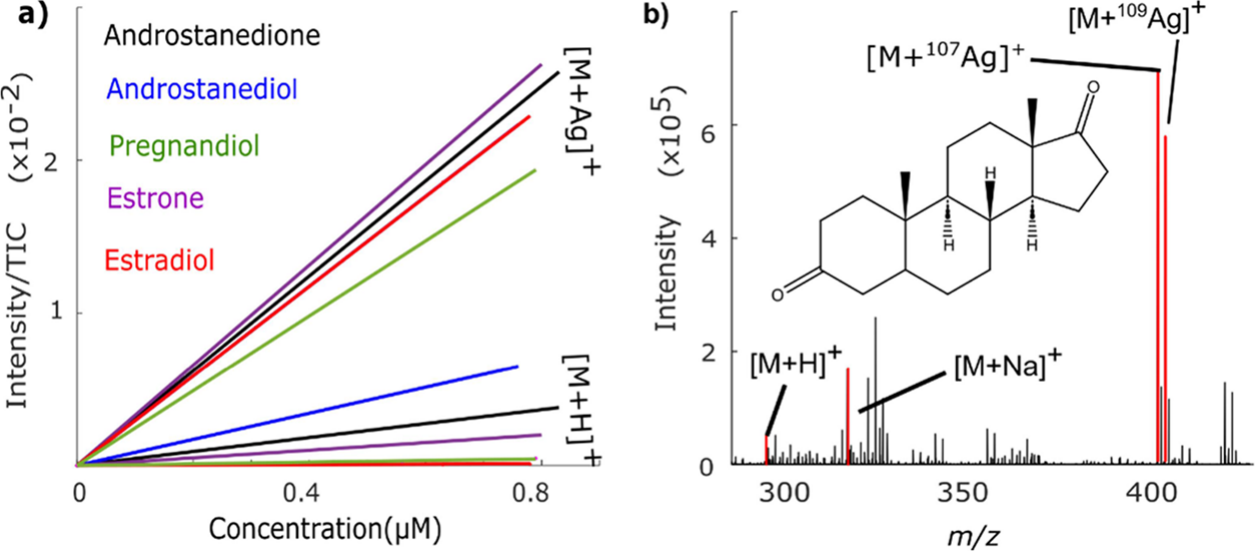
Silver cationization increases the sensitivity of both saturated and unsaturated steroids. (a) Sensitivity evaluation for selected saturated and unsaturated C18, C19, and C21 steroid standards as [M +^107^Ag]^+^ and [M + H]^+^ ions. The evaluation covered the concentration range of 0–0.9 μM. (b) Mass spectrum of 5α-androstan-3,17-dione with labeled adducts.

Steroids generally form protonated adduct ions ([M + H]^+^) and metal adduct ions such as [M + Na]^+^ and [M + K]^+^ from naturally occurring salts in ESI-MS (Figures S1 and S2). Steroid standards can be deliberately cationized with Ag^+^ by adding an optimized concentration of AgNO_3(aq)_ to the ESI solvent. To investigate any enhancement in sensitivity, a series of estrogens, androgens, and progestogens were analyzed in positive ion mode as [M + H]^+^ and [M + Ag]^+^. Figure 1a represents the respective results, where when using the slope of the calibration curve as a measure of analytical sensitivity, it is evident that silver significantly improves sensitivity. Specifically, the response curve data shown in Table S1 show a 2–150 times increase in sensitivity for [M + Ag]^+^ when compared to [M + H]^+^ for 5α-androstanedione, 3α-androstanediol, pregnanedione, estrone, and 17β-estradiol. It should be noted that this improvement in sensitivity occurs despite the two naturally occurring silver isotopologues, 52% ^107^Ag and 48% ^109^Ag that split the peak. However, the unique isotopic pattern of [M +^107^Ag]^+^ and [M +^109^Ag]^+^ benefits spectral interpretation for rapidly identifying silver adducts. The finding of increased sensitivity of [M + Ag]^+^ correlates well with previous reports on enhanced signals of unsaturated prostaglandins^19^ and fatty acids,^20^ upon Ag^+^ complexation with C=C bonds in MS.

In addition to complexation with C=C, Ag^+^ complexes more intensely with C=O and C–OH moieties compared to H^+^ and Na^+^. This is evidenced by the intense signal of saturated steroids 5α-androstan-3,17-dione and 5α-androstane-3α,17-diol as [M + Ag]^+^ (Figures 1b and S3, respectively). Specifically, both the saturated 5α-androstan-3,17-dione with C=O moieties and the saturated 5α-androstane-3α,17-diol with C–OH moieties show strong [M + Ag]^+^ peaks. Thus, silver cationization can be achieved on molecules containing either C=C, C=O, or C–OH and is therefore not confined by the inherent limitation of derivatizing reagents such as Girard T, which is commonly used to derivatize steroids with C=O to increase sensitivity in MALDI MSI workflows. The increased sensitivity and ability to coordinate with various bonds demonstrate the applicability and versatility of Ag^+^ cationization for MSI of steroids.

### Fragmentation of Steroids as Silver Adducts Increases Specificity

**Figure 2:**
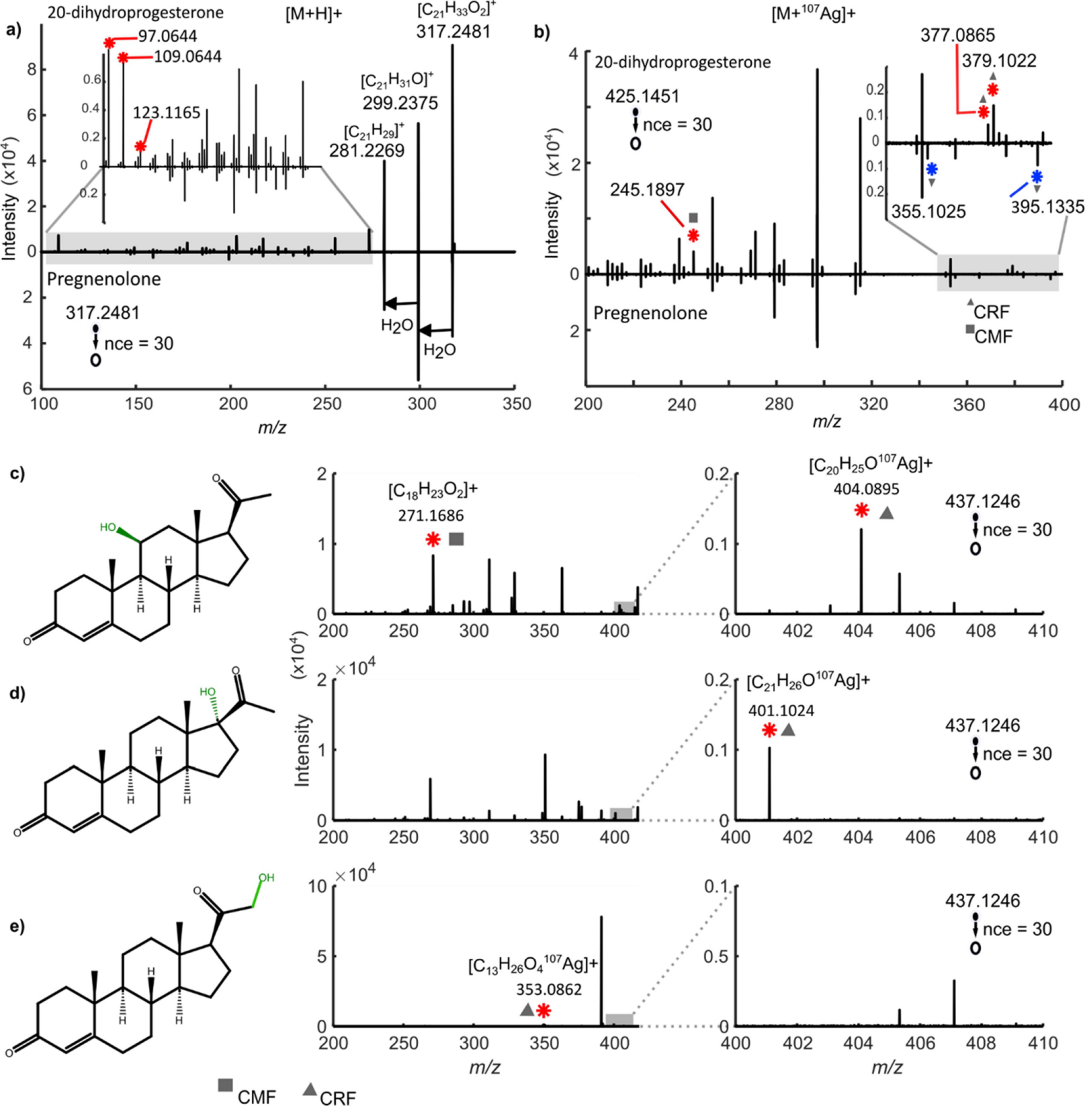
Silver adduct fragmentation increases specificity. (a) MS^2^ spectra of the protonated adduct of 20α-dihydroprogesterone produce unique product ions at *m*/*z* 109.0644 and 123.1165 (red star), while fragmentation of the protonated adduct of pregnenolone (inverted) produces isomeric product ions only. (b) MS^2^ spectra of silver adduct of 20α-dihydroprogesterone produce unique product ions at *m*/*z* 245.1897, 377.0865, and 379.1022 (red stars) and pregnenolone (inverted) at *m*/*z* 355.1021 and 395.113 (blue stars). Fragmentation in MS^2^ of [M + ^107^Ag]^+^ of three hydroxyprogesterone regioisomers that differ in the position of the hydroxyl group produces DPIs (c–e). (c) 11-hydroxyprogesterone, (d) 17-hydroxyprogesterone, and (e) 21-hydroxyprogesterone. Product ions generated via CRF and CMF are denoted by triangles and squares, respectively. Note: other major *m*/*z* values for hydroxyprogesterone are given in Table S2.

Annotation by direct MS techniques, such as MSI, requires high-resolution mass measurements and high-quality fragmentation spectra. Our results show that silver cationization and subsequent CID enhance fragmentation specificity. For example, the regioisomers pregnenolone and 20α-dihydroprogesterone, which differ in the position of three functional groups, show improved specificity when fragmented in CID as [M +Ag]^+^ compared to [M + H]^+^, [M + H–H_2_O]^+^, [M + Li]^+^, [M + Na]^+^, and [M + K]^+^. Specifically, [M + Li]^+^ and [M + H–H_2_O]^+^ mainly loose water in MS^2^, while no product ions were detected for [M + Na]^+^ and [M + K]^+^ (Figures S4 and S5). Fragmentation of pregnenolone [M + H]^+^ produces isomeric product ions only, while the three unique product ions of 20α-dihydroprogesterone [M + H]^+^ are generally found for all steroids in the subgroup “3-oxo-4-ene” at *m*/*z* 97.0644, 109.0644, and 123.1165 when fragmented in CID (Figure 2a).^21^ In contrast, [M + Ag]^+^ fragmentation in MS^2^ produces regioisomer-specific DPIs for both 20α-dihydroprogesterone and pregnenolone. Specifically, three DPIs are observed for 20α-dihydroprogesterone at *m*/*z* 245.1897, 377.0865, and 379.1022, and two DPIs are observed for pregnenolone at *m*/*z* 355.1095 and 395.1335 (Figure 2b). When these ions are further fragmented as [M + Ag–H_2_O]^+^ in MS^3^, the number of DPIs increases to five and six, respectively (Figure S6). Overall, the CID fragmentation of [M + Ag]^+^ in MS^2^ provides unique product ions that enable annotation at the regioisomeric level with MS^*n*^ alone.

An increase in structural similarity suggests increased difficulty in separating and identifying steroid regioisomers by MS^*n*^ alone. The regioisomers 11-hydroxyprogesterone, 17-hydroxyprogesterone, and 21-hydroxyprogesterone only differ by the position of the C–OH group, which is either carbon 11, 17, or 21, respectively (Figure 2c,d). Moreover, they are all 3-oxo-4-ene steroids and thus cannot be differentiated by the fragmentation of protonated adducts (Figure S7). Nevertheless, upon CID fragmentation of [M + Ag]^+^ in MS^2^, DPIs are successfully detected from each hydroxyprogesterone regioisomer (Figure 2c,d). Specifically, two DPIS are observed for 11-hydroxyprogesterone at *m*/*z* 271.1686 and 404.0895, while one DPI each for 17-hydroxyprogesterone and 21-hydrohyprogesterone at *m*/*z* 401.1024 and 353.0862, respectively. Even more challenging is to annotate epimers such as 11α-hydroxyprogesterone and 11β-hydroxyprogesterone, which differ only by the orientation of the hydroxyl group (Figure S2). However, upon MS^2^ of their [M + Ag]^+^, a DPI of the β structure is detected that can be used to differentiate the two epimers (Figure S8). Thus, the CID of [M + Ag]^+^ also generates structurally unique product ions of highly similar steroid regioisomers by MS alone.

Product ions generated upon fragmentation of silver adduct steroids are formed via both charge retention/charge remote fragmentation (CRF) and charge migration fragmentation (CMF) (Scheme S1). In CRF, Ag^+^ is retained on the product ion in contrast to CMF where Ag^+^ is lost during fragmentation leaving the charge on a carbon atom of the product ion. Previously, CRF of steroids in low energy CID has been reported for steroid conjugates that are detected in negative ESI mode.^22^ This is because the conjugate, either glucuronate or sulfate, maintains the fixed stable charge on the molecule that is required for CRF to occur. In negatively charged steroid conjugates, both CRF and CMF occur, which complicates the interpretation of the fragmentation pathway. In the CID of Ag^+^ cationized steroid adducts, both CRF and CMF also occur. However, the unique isotopic pattern of ^107^Ag^+^ and ^109^Ag^+^ simplifies mass spectral interpretation by providing a clear difference between CRF and CMF. Additionally, since Ag^+^ provides the stable charge needed for CRF to occur in tandem MS, silver cationization can be extended to neutral steroids. Overall, silver cationization provides a flexible alternative to assessing steroid fragmentation pathways.

### Silver Adduct Fragmentation Patterns Can Be Generalized

**Figure 3:**
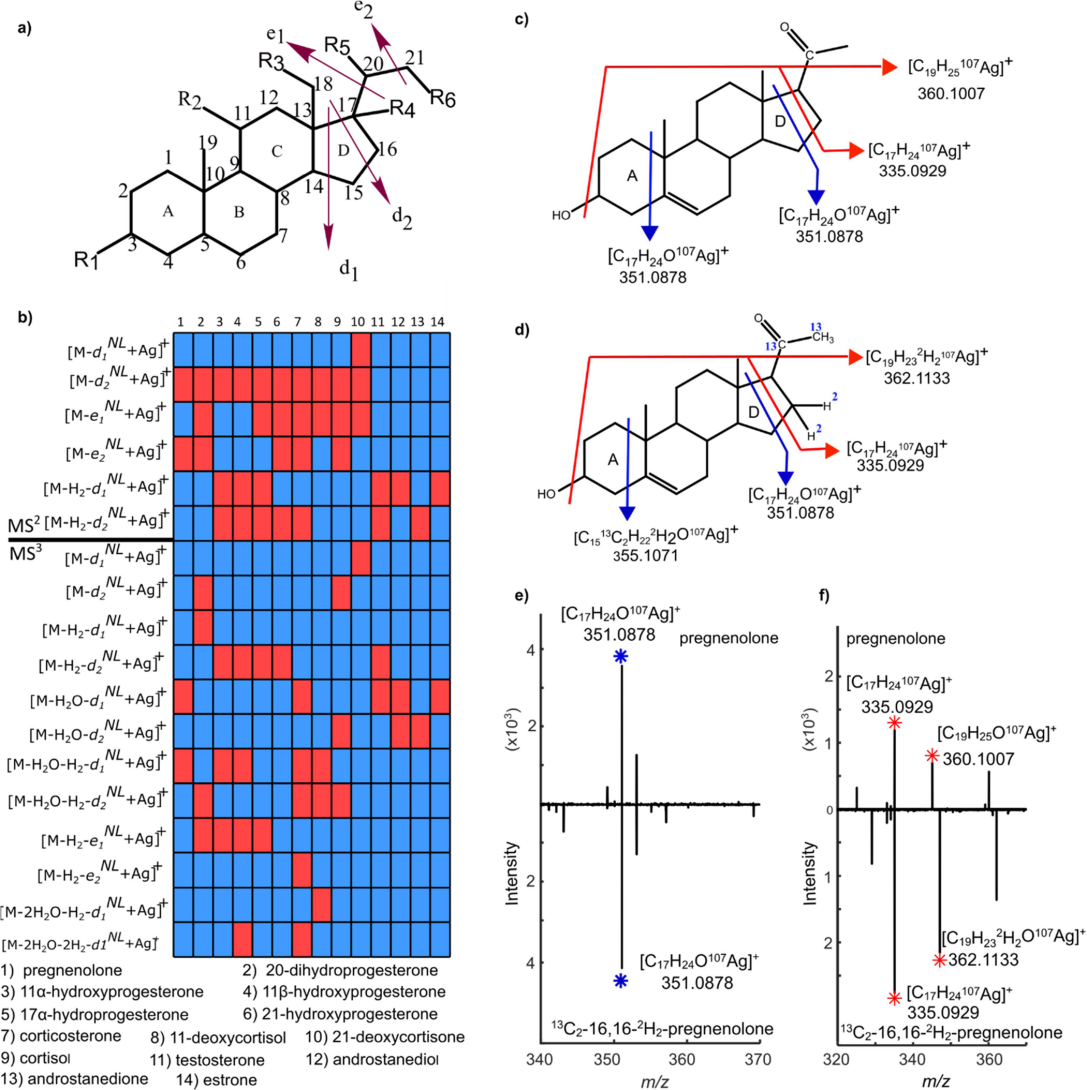
Predictable DPIs are formed in MS^2^ upon fragmentation of steroids as [M + Ag]^+^. (a) General structure of steroids with rings A–D together with the e-side chain (C20 and C21), the hydroxy, and/or ketone functional group positions are indicated by R1 to R6. The arrows mark the general fragmentation pattern of C21 steroids as silver adducts in CID with d_1_, d_2_, e_1_, and e_2_ as fragment ions resulting from CRF. (b) Fragmentation of 14 different steroids (1–14) via CID in MS^2^ and MS^3^ results in CRF with neutral loss (NL) from the d-ring and the e-side chain, denoted d_1_^NL^, d_2_^NL^, e_1_^NL^, and e_2_^NL^. The presence and absence of product ions for steroids 1–14 are indicated in red and blue, respectively. (c)Molecular structure of the pregnenolone molecule. Blue arrows: two potential sites of fragmentation for product ion at *m*/*z* 351.0878 ([C_17_H_24_O^107^Ag]^+^) in MS^2^. Red arrows: possible sites of fragmentation resulting in production ions at *m*/*z* 335.0929 ([C_17_H_24_^107^Ag]^+^) and 360.1007 ([C_19_H_25_O^107^Ag]^+^) in MS^3^. (d) Molecular structure of ^13^C_2_-16,16-^2^H_2_-pregnenolone molecule with arrows as described in (c). (e) MS^2^ mass spectra of pregnenolone and ^13^C_2_-16,16-^2^H_2_-pregnenolone (inverted) both show the dissociation of the d-ring with the product ion at *m*/*z* 351.0878. f) MS^3^ spectra of pregnenolone and ^13^C_2_-16,16-^2^H_2_-pregnenolone (inverted) confirm the fragmentation site of the d-ring (*m*/*z* 335.0929) and the e-side chain by a shift from *m*/*z* 360.1007 to 362.1133 by the two deuterated atoms of ^13^C_2_-16,16-^2^H_2_-pregnenolone.

We have identified a general CRF pattern for [M + Ag]^+^ of steroids by investigating several steroid standards. This pattern includes the cleavage of C–C bonds in the d-ring and e-side chain when silver adduct steroids are subjected to CID in MS^2^ or MS^3^ (Figure 3a). Specifically, product ions generated by CRF result from a neutral loss (NL) of the partial d-ring or e-side chain from C–C cleavages, indicated by arrows labeled d_1_, d_2_, e_1_, and e_2_. For example, the MS^2^ product ion at *m*/*z* 377.0865 for 20α-dihydroprogesterone shown in Figure 2b is generated by the NL arising from the fragmentation at e_1_, which is between C_17_ and C_20_ in Figure 3a, and therefore denoted [M-e_1_^NL^ + ^107^Ag]^+^. The presence and absence of product ions with NL based on the dissociation of C–C bonds at d_1_, d_2_, e_1_, and e_2_ are indicated in red and blue, respectively, for 14 different silver adduct steroids subjected to fragmentation in MS^2^ or MS^3^ (Figure 3b, above and below the line, respectively). For example, DPIs for pregnenolone in MS^2^ correspond to [M-*d*_2_^NL^ + ^107^Ag]^+^ and [M-e_2_^NL^ + ^107^Ag]^+^, and in MS^3^ to [M-H_2_O-*d*_1_^NL^ + ^107^Ag]^+^ and [M-H_2_O–H_2_-d_1_^NL^ + ^107^Ag]^+^ (labeled 1 in Figure 3b, while the plausible molecular structure of d_1_^NL^, d_2_^NL^, and e_1_^NL^ for the pregnenolone molecule is given in Figure S9). Comparison of NL from the d-ring and the e-side chain for all 14 steroid standards in the diagram shows the high specificity that comes with CRF in CID of steroids as silver adduction.

Validation of the DPIs generated by dissociation of C–C bonds in the d-ring and e-side chain was conducted using an isotopically labeled standard of pregnenolone (^13^C_2_-16,16-^2^H_2_-pregnenolone). Specifically, the product ion at *m*/*z* 351.0878 in the MS^2^ spectrum of pregnenolone, denoted [M-*d*_2_^NL^ + ^107^Ag]^+^, could potentially also arise from the dissociation of the C–C bond between C_1_–C_10_ and C_4_–C_5_ of the a-ring (blue arrows in Figure 3c,d). However, the identical product ions at *m*/*z* 351.0878 and 335.0929 of both pregnenolone and the labeled standard confirm that the cleavage site is in the d-ring (Figure 3e,f). Furthermore, the increased *m*/*z* from 360.1007 to 362.1133 that correspond to the two deuterium atoms in the d-ring of the labeled pregnenolone confirm the dissociation of the C–C bond in the e-side chain (Figure 3f). A plausible mechanism for the d-ring fragmentation of pregnenolone is given in Scheme S2. Notably, the product ions arising from CRF are observed only with CID and not with higher-energy collision-induced dissociation (HCD) (Figure S10). Thus, DPIs from the d-ring and e-side chain of steroid isomers can only be generated from silver adduct steroids that are subjected to CID.

Functional groups giving rise to steroid isomers are preferentially situated around the d- and e-side chains, making the DPIs highly important. Thereby, our identified and reproducible fragmentation pathway can be used to predict steroid structures with regioisomeric specificity even from nontargeted steroids. For example, in a sample, the two regioisomers 11-deoxycortisol and corticosterone, which differ only by one hydroxyl group at position 11 or 17, respectively, were tentatively identified based on the dissociation between C_13_–C_17_ and C_14_–C_15_ of the d-ring (Figure S11). This was subsequently confirmed by analysis of the respective standards using the fragmentation pathway (Figures S12 and S13). Overall, the consistent CRF pattern of steroid isomers in MS^2^ enables direct and unique prediction of the steroid structure with regioisomeric specificity.

### Nano-DESI Imaging of Steroids as Silver Adducts

The ease of silver incorporation into solvents in combination with the afforded increase in sensitivity, the ability to coordinate with various bonds, and the increased specificity in tandem MS suggest the applicability for MSI of steroids. For spatial mapping of thin tissue sections, MSI of Ag^+^ adduct ions can be readily performed with PA nano-DESI without modifying the sample.^19^ Nano-DESI is a surface sampling technique that extracts analytes from tissue surfaces by a localized liquid bridge (Scheme S3).^23,24^ Briefly, the analytes are desorbed from the tissue surface into a liquid bridge flowing between two fused silica capillaries positioned in front of the mass spectrometer. Subsequently, the desorbed analytes are transported via the secondary capillary by the Venturi effect to the inlet of the mass spectrometer for pneumatically assisted electrospray ionization. By constantly moving the sample under the localized liquid bridge, data are continuously acquired for the consequent construction of 2-D maps visualizing analyte distribution in the tissue. Each pixel on the constructed 2-D map corresponds to the intensity of a selected ion from each scan event by data acquisition. In nano-DESI, challenging analytes can be targeted by the addition of reagents for reactive chemistry.^25,26^

**Figure 4:**
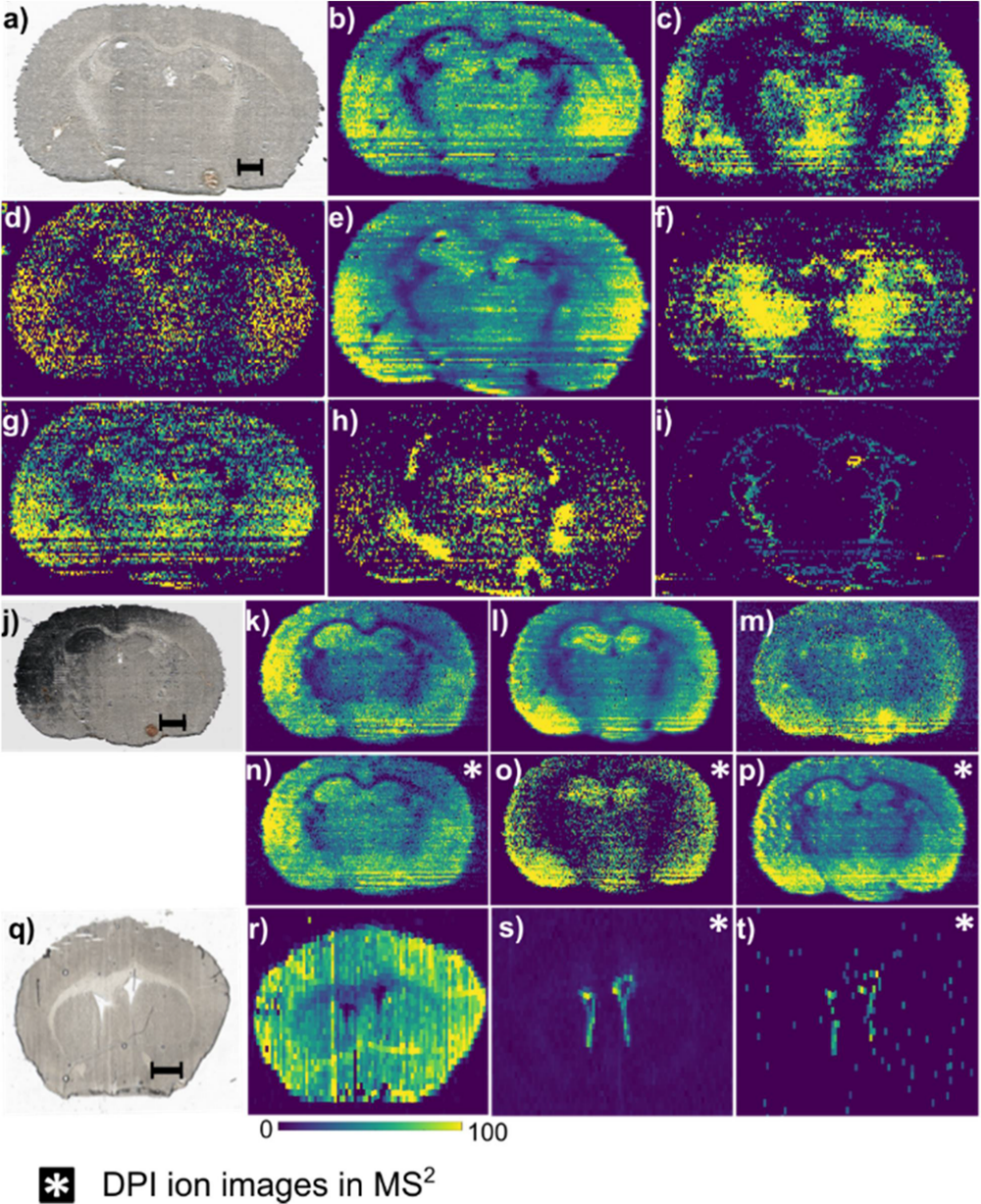
Silver cationization of steroids with PA nano-DESI provides high sensitivity and specificity for MSI. (a) Optical image of the imaged mouse brain section. Putatively annotated ion images of *m*/*z* corresponding to steroids as [M + ^107^Ag]^+^ in MS^1^ (b) estradiol (*m*/*z* 379.0827), (c) hydroxyestradiol/estriol (*m*/*z* 395.0776), (d) 7-hydroxydehydroepiandrosterone (*m*/*z* 411.1089), (e) pregnanolone *(m*/*z* 425.1601), (f) aldosterone/cortisone (*m*/*z* 467.0988), (g) androstanediol (*m*/*z* 399.1453), (h) pregnenolone/5α-dihydroprogesterone (*m*/*z* 423.1453), and i) estetrol *(m*/*z* 411.0726). (j) Optical image of the mouse brain section imaged in (k–p) MS^1^ ion images of mass channels corresponding to (k) estradiol (*m*/*z* 379.0827), l) pregnanolone *(m*/*z* 425.1601), and (m) androstanediol (*m*/*z* 399.1453). MS^2^ ion images of DPIs corresponding to (n) estradiol, ([M + Ag-d_1_]^+^; 335.0544), (o) pregnanolone ([M + Ag–H_2_O-*d*_1_]^+^;337.1085), and (p) androstanediol ([M + Ag–H_2_-d_1_]^+^; 335.0939). (q) Optical image of the mouse brain section imaged in r–s. (r) Ion image of the precursor ion at *m*/*z* 423.1451. MS^2^ ion images of DPIs showing (s) 16-pregnenolone ([M + Ag + H_2_-d_1_]^+^; 341.1034) and (t) 5-dihydroprogesterone/3-dihydroprogesterone ([M + Ag–H_2_-d_1_]^+^;337.0721). Note: all images are constructed from raw data (absolute intensities).

In an experiment, a ^107^Ag^+^ containing a mixture of CH_3_CN/CH_3_OH (9:1 v/v) was propelled through the nano-DESI probe while a mouse brain tissue section was moved under the probe for silver-doped PA nano-DESI MSI. The acquired data show the detection of numerous neurosteroids from tissue as Ag^+^ adducts that were not detectable without silver (Figure S14a–g). In comparison, extraction solvent without Ag^+^, that is, CH_3_CN/CH_3_OH (9:1 v/v) with 0.1% formic acid, either had high interferences from solvent peaks or low and incoherent signal intensities for the corresponding [M + H]^+^ and [M-H_2_O + H]^+^ ions. (Figure S14h–u). Subsequently, the 13 ion images of putatively annotated silver-cationized steroids show distinct distributions in the brain tissue (Figures 4b–i and S15), which agree with previous mRNA expression studies.^27^ In particular, estradiol, hydroxyestradiol/estriol, 7-hydroxydehydroepiandrosterone, and pregnanolone (Figure 4b–e) are mainly distributed in the gray matter. Aldosterone/cortisone is most abundant in the thalamus and white matter, while androstanediol is distributed throughout the tissue (Figure 4f,g). Additionally, the data reveal intricate distributions of estetrol and pregnenolone and its isomers to subregions of the white matter (Figure 4h,i). Our silver-doped PA nano-DESI approach enables the first reported visualization of these endogenous steroid distributions in the brain.

Despite the excitement of simultaneously visualizing multiple steroids in brain tissue, each *m*/*z* may contain multiple isobars and isomers that are unresolved, even with high-resolution mass measurements. Isobaric and isomeric endogenous species have been previously mapped in tissue by combining MSI with MS^2^, MS^3^, or MS^4^.^13,26,28−33^ Here, we demonstrate imaging of steroids with isomeric precision by their distinctive CRF pattern using silver-doped PA nano-DESI and report their distributions in mouse brain tissue (Figure 4j–t). The similar distribution of the precursor and DPI for estradiol and pregnanolone suggests that this isomer is the major analyte in the mass channel (Figure 4k–l and n–o). However, the DPI image of androstanediol shows more distinct features than the precursor ion image (Figure 4m,p), which suggests multiple isobars or isomers in the precursor ion mass channel. Moreover, the DPIs of the regioisomers 16-pregnanolone and 5-dihydroprogesterone (Figure 4s,t) show specific localizations around the lateral ventricles of the mouse brain that cannot be inferred from the precursor image (Figure 4r). It is imperative to separate these regioisomers since the 5-dihydroprogesterone is a neurosteroid modulator for the GABA receptor in contrast to its less studied regioisomer.^34^ Thus, imaging the DPI to reveal the distribution of individual isomers will provide precise information about the importance of the distinct isomers in the respective morphological regions. Overall, the developed method provides a novel and vital tool for steroid research and biological understanding.

## Conclusions

In this study, we introduce a method for detecting and imaging steroid regioisomers in biological samples using MSI. The method is highly selective and does not require prior derivatization, chromatographic separation, or instrumental modification. By adding silver and performing CID of steroids as [M + Ag]^+^ in MS^2^, the d-ring and e-side chain fragment through CRF produce DPIs that can accurately annotate steroid and steroid isomers. Applying the method with nano-DESI imaging maps previously unknown distributions of some endogenous steroids in brain tissue. These findings suggest a promising future for uncovering the role of steroid isomers in biological processes and exploiting their potential as biomarkers and therapeutics.

## Supporting Information Available

The general structure of steroids including C18, C19, and C21 steroids; tandem mass spectra of protonated, lithiated, and silver adducts of steroid molecules; plausible neutral loss and fragment ion structure upon silver adduct fragmentation; nano-DESI extracted ion chronograms for line scans comparing silver and protonated adducts; schematics CMF and CRF; schematics for the plausible fragmentation mechanism; schematics of PA nano-DESI; and sensitivity of silver adducts compared to protonated adducts (PDF)
