# Supplementary material for: Revealing Structure and Localization of Steroid Regioisomers through Predictive Fragmentation Patterns in Mass Spectrometry Imaging
Source: Anal Chem. 2023 Nov 17;95(48):17843–50. doi: 10.1021/acs.analchem.3c03931 (PMC10701710; doi:10.1021/acs.analchem.3c03931)
Supplement: Supplementary file 1 — ac3c03931_si_001.pdf [file ac3c03931_si_001.pdf]

Supporting Information

Revealing structure and localization of steroid regioisomers through predictive fragmentation patterns in mass spectrometry imaging

Varun V. Sharma,† and Ingela Lanekoff †,*

† Department of Chemistry – BMC, Uppsala University, Husargatan 3, 751 23 Uppsala, Sweden

*Corresponding author: ingela.lanekoff@kemi.uu.se

Table of Contents

Figures S2

Schemes S12

Tables S14

Figures

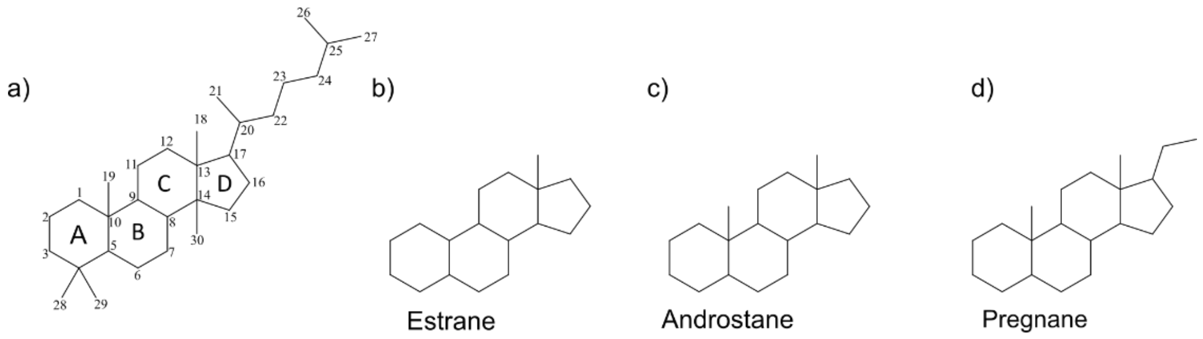

Figure S1 a) General structure of steroid molecules represented by Lanostane molecule (30 carbon atoms) with IUPAC-approved numbering and lettering. The common skeletal structure consists of four core ring systems (A, B, C & D) composed of 17 carbon atoms. The general structure of the three endogenous steroid groups a) estrogens (18 carbon atoms), c) androgens (19 carbon atoms) d) progestogens (21 carbon atoms)

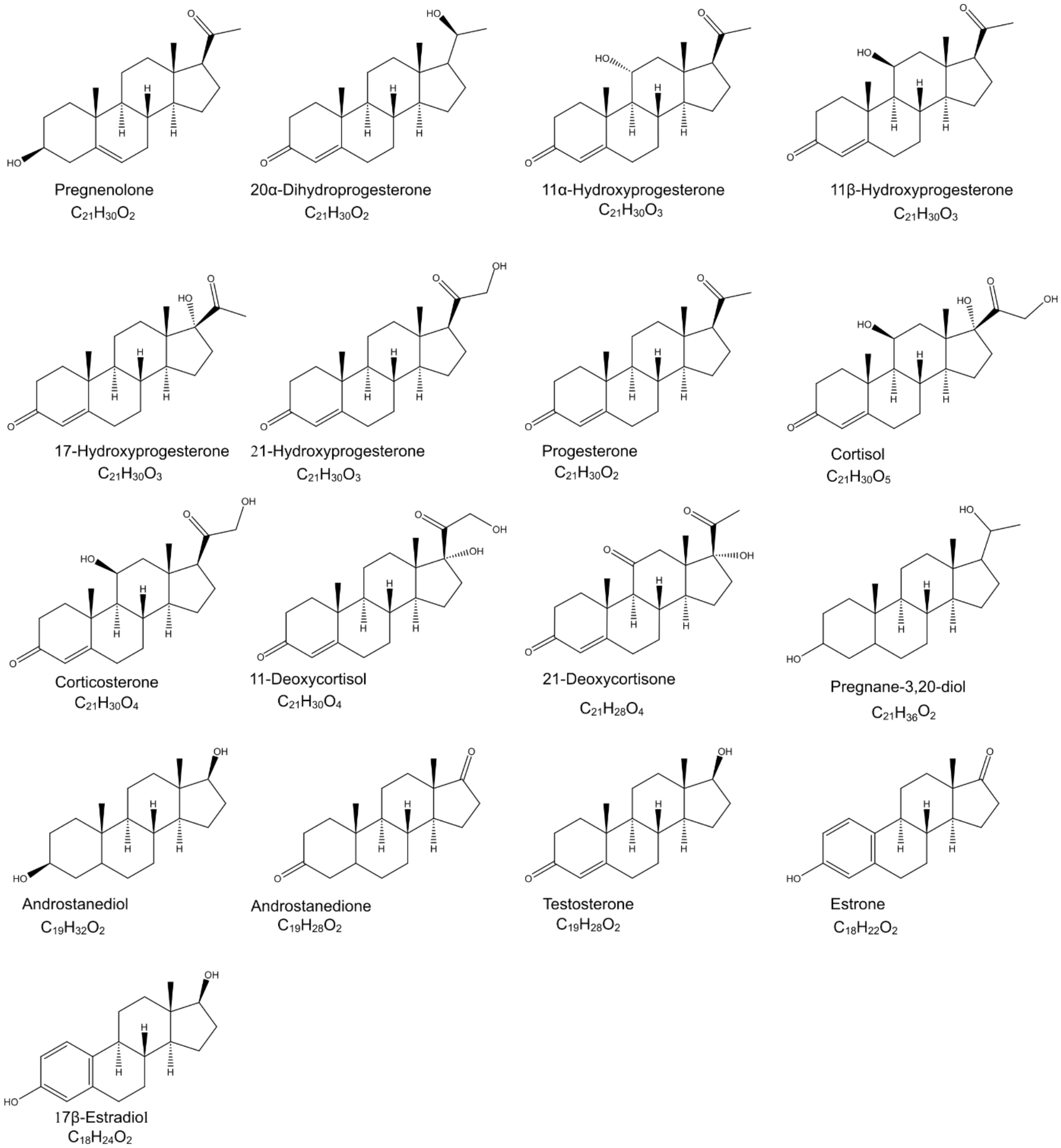

Figure S2 Structures and molecular formulas of steroid standards investigated in this study.

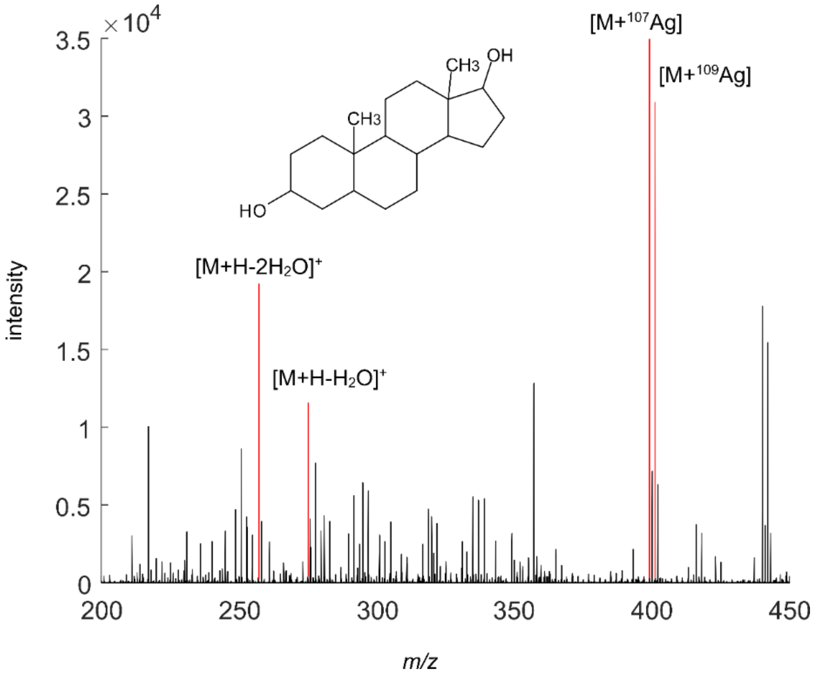

Figure S3. Silver cationization of saturated steroid androstanediol at 0.5 µM in MS^1. Peaks related to the cationization of androstanediol are marked in red. Silver forms intense $[M+^{107}Ag]^+$ and $[M+^{109}Ag]^+$ peaks with androstanediol compared to in source fragmented ions of protonated adducts ($[M+H-H_2O]^+$ & ($[M+H-2H_2O]^+$). Androstanediol does not form protonated or sodiated adducts.

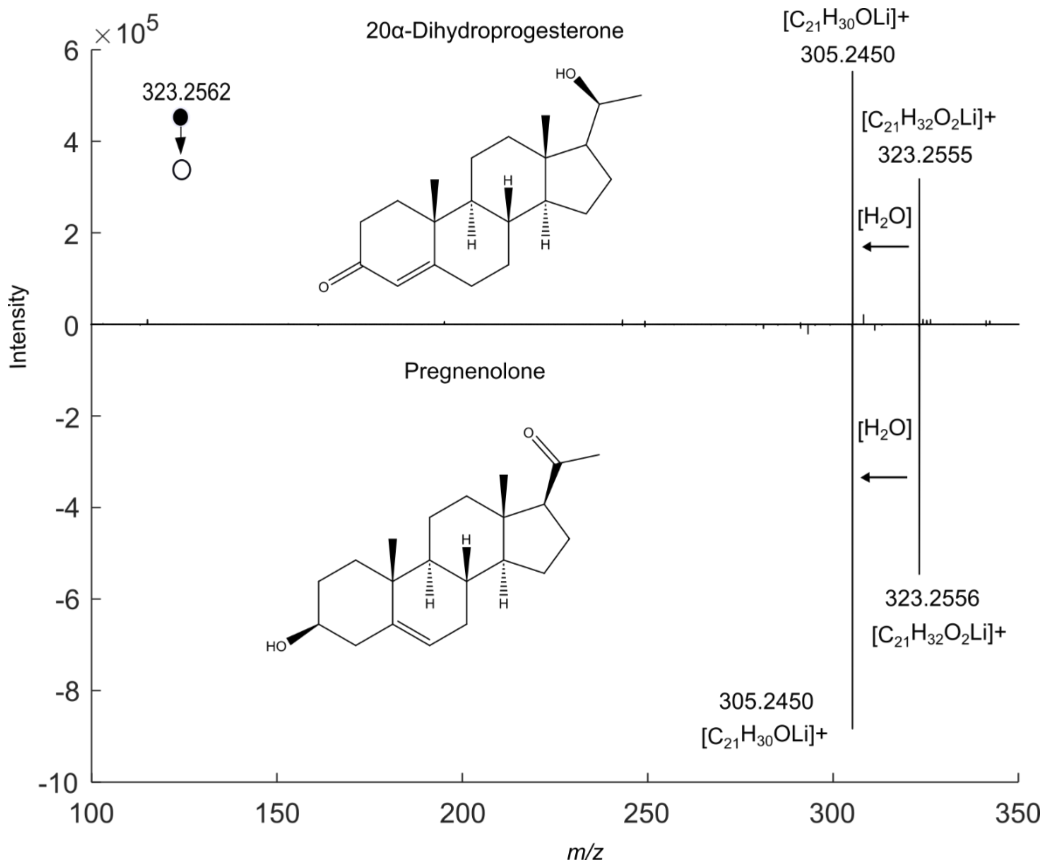

Figure S4 Lithium adducts predominantly undergo loss of H_2O in CID for the regioisomers 20α-dihydroprogesterone and pregnenolone. 10 µM of both regioisomers were directly infused into Orbitrap Velos Pro at a flow rate of 10 µl/min and fragmented at a collision energy of 35 NCE at a resolution of 100 000 (*Δm/m* at *m/z* 400)

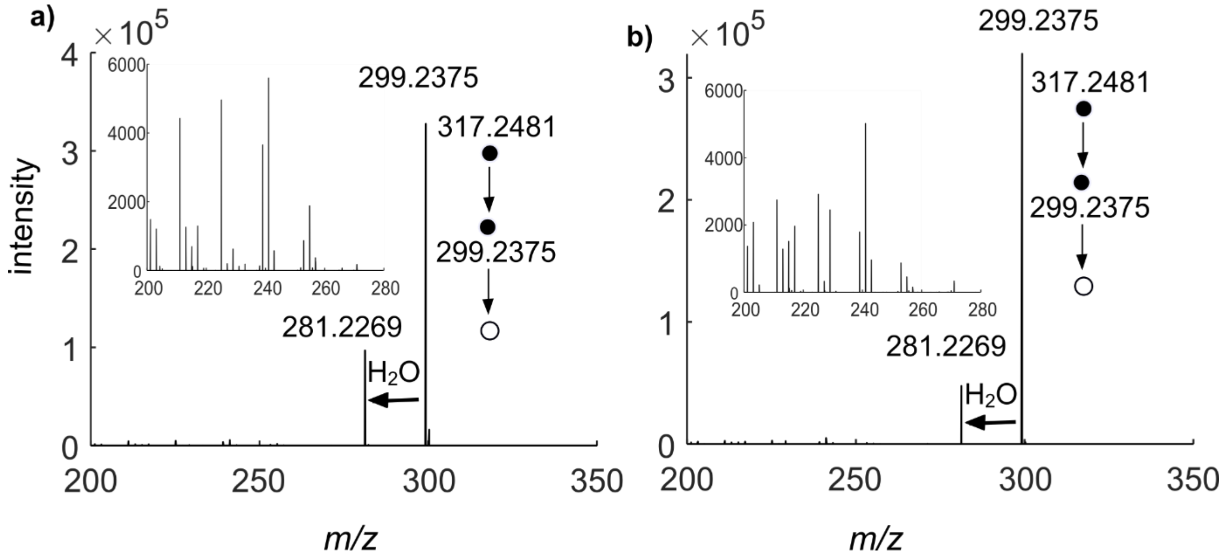

Figure S5 Fragmentation of 50 µM protonated adducts of pregnenolone and 20α-dihydroprogesterone as $[M+H-H_2O]^+$ in CID predominantly results in isomeric product ions due to the loss of H_2O molecules. a) MS^3 spectra from the fragmentation of $[Pregnenolone+H-H_2O]^+$. b) MS^3 spectra from the fragmentation of $[20α-dihydroprogesterone +H-H_2O]^+$. The fragment ions in MS3 spectra in the *m/z* range of 200 to 280 are also isomeric although the intensities vary. Both regioisomers were directly infused into Orbitrap Velos Pro at a flow rate of 10 µl/min and fragmented at a collision energy of 20 NCE at a resolution of 100 000 (*Δm/m* at *m/z* 400)

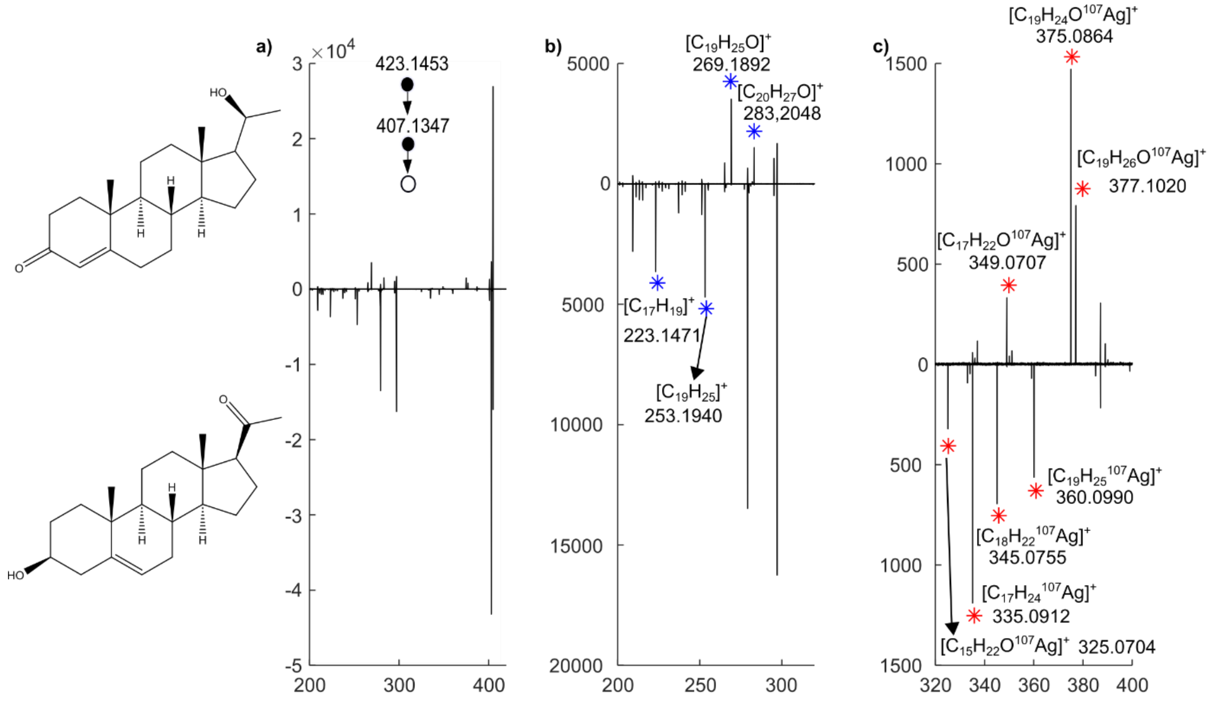

Figure S6. Diagnostic product ions are formed in MS³ upon fragmentation of Ag⁺ cationized steroids. a) MS³ spectrum of 20α-dihydroprogesterone and inverted mass spectrum of pregnenolone, both at 10 µM b) MS³ spectrum of 20α-dihydroprogesterone and inverted mass spectrum of pregnenolone zoomed in on *m/z* range 200 – 320 where DPIs form via CMF with Ag+ ion being lost from product ions during fragmentation. MS³ spectrum of 20α-dihydroprogesterone and inverted mass spectrum of pregnenolone zoomed in on *m/z* range 320 – 400 where DPIs form via CRF with Ag+ ion being retained in product ions during fragmentation. Both regioisomers were directly infused into Orbitrap Velos Pro at a flow rate of 10 µl/min and fragmented at a collision energy of 20 NCE at a resolution of 100 000 (*Δm/m* at *m/z* 400). All *m/z* values are with ±5 ppm of the theoretical value.

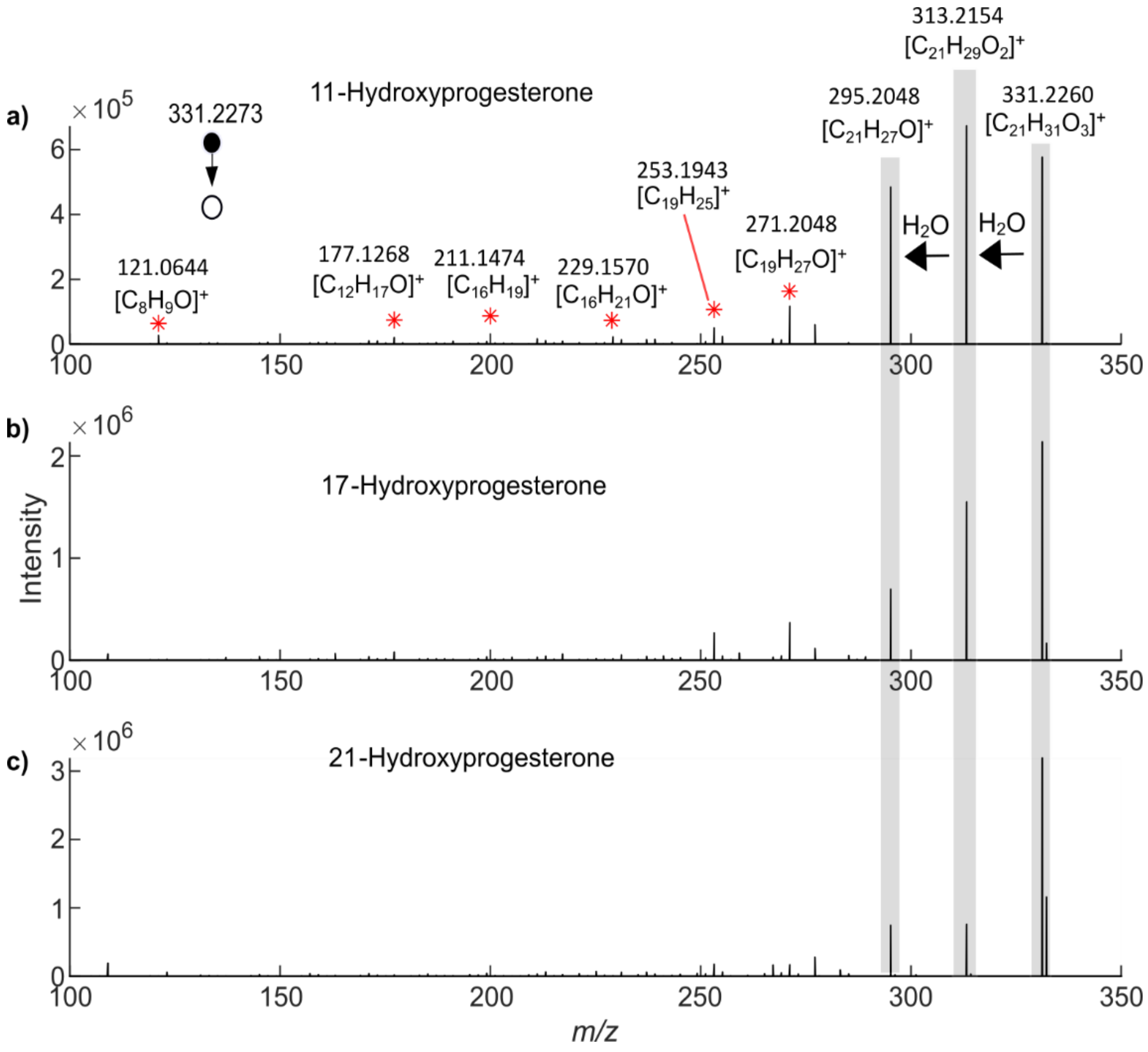

Figure S7 Protonated adducts of three hydroxyprogesterone's fragment in CID to give isomeric product ions in MS^2. a) MS2 mass spectrum of 11-hydroxyprogesterone b) MS^2 mass spectrum of 17-hydroxyprogesterone c) MS^2 mass spectrum of 21-hydroxyprogesterone. All regioisomers were directly infused into Orbitrap Velos Pro at a flow rate of 10 µl/min and fragmented at a collision energy of 20 NCE at a resolution of 100 000 ($\Delta m/m$ at m/z 400). All m/z values are with ±5ppm of the theoretical value.

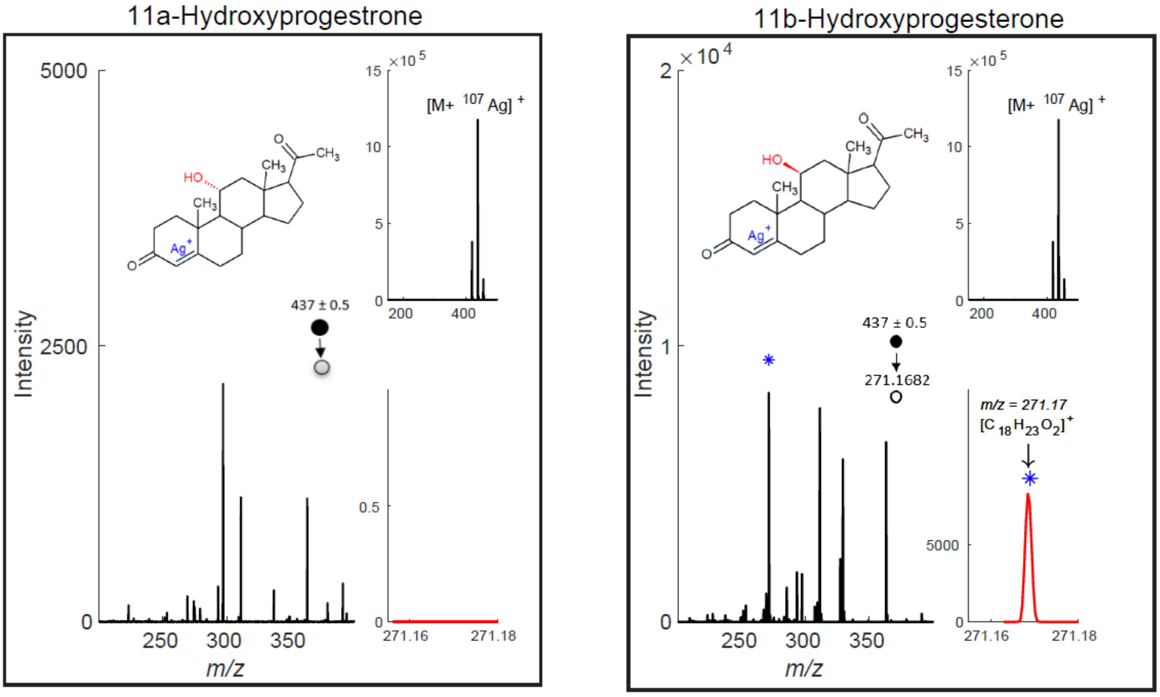

Figure S8. Fragmentation of 11βOHP produces a diagnostic product ion at m/z = 271.1710 differentiating it from its epimer 11αOHP. All regioisomers were directly infused into Orbitrap Velos Pro at a flow rate of 10 µl/min and fragmented at a collision energy of 20 NCE at a resolution of 100 000 ($\Delta m/m$ at *m/z* 400). All *m/z* values are with ±5ppm of the theoretical value.

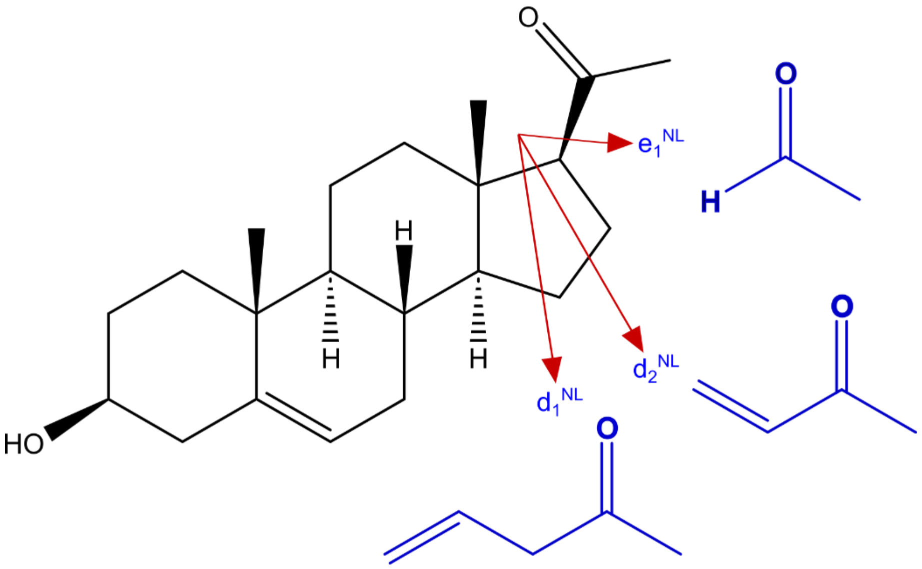

Figure S9 Plausible molecular structure of neutral loss fragments upon fragmentation of argenated adduct of pregnenolone molecule with CID.

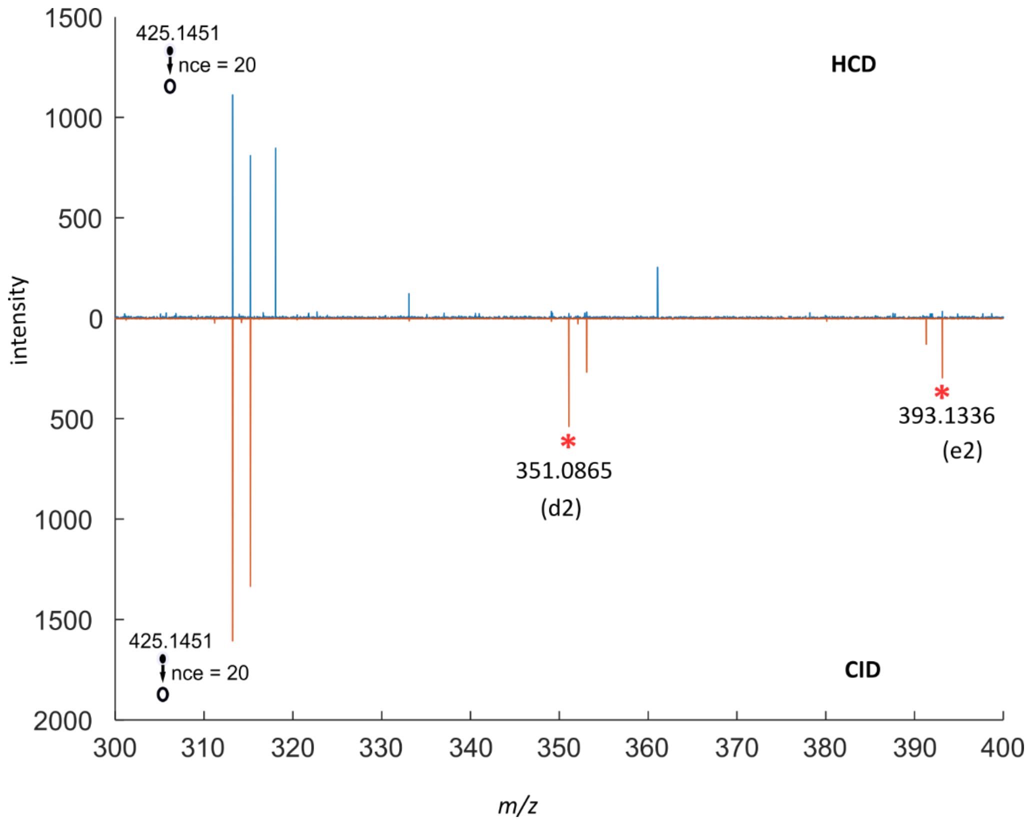

Figure S10. Fragmentation of d-ring and e-side chain by CRF only occurs by CID and not by HCD. MS^2 spectra of pregnenolone fragmented by HCD (top) and CID (inverted).

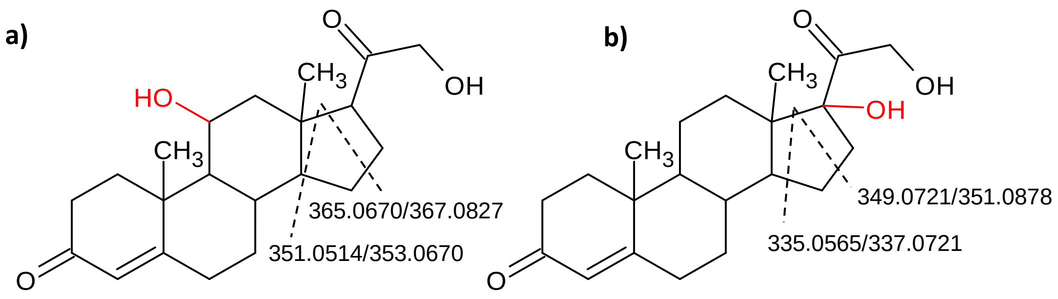

Figure S11. Predicted fragmentation that enables separation of 11DOC and CORT. a) *m/z* at 365.0670 and 367.0670 corresponds to the fragment ion $C_{17}H_{22}O_2{}^{107}Ag$ and $C_{17}H_{24}O_2{}^{107}Ag$ of corticosterone. b) *m/z* at 349.0721 and 351.0878 corresponds to $C_{17}H_{22}O_2{}^{107}Ag$ and $C_{17}H_{24}O_2{}^{107}Ag$ of 11-deoxycortisol.

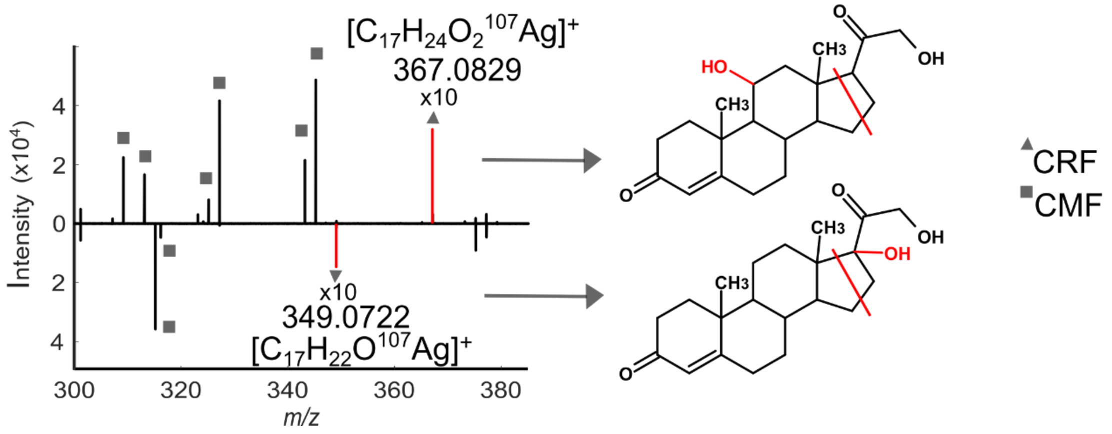

Figure S12 CID of the two regioisomers corticosterone and 11-deoxycortisol as $[M+Ag]^+$ induce c-c bond dissociation in the d-ring producing isomer specific fragment ions in MS^2 with less than 2 ppm mass difference compared to the theoretically predicted *m/z* values. Corticosterone produces a fragment ion at *m/z* = 367.0829 and 11-deoxycortisol produces a fragment ion at *m/z* = 349.0722 corresponding d2.

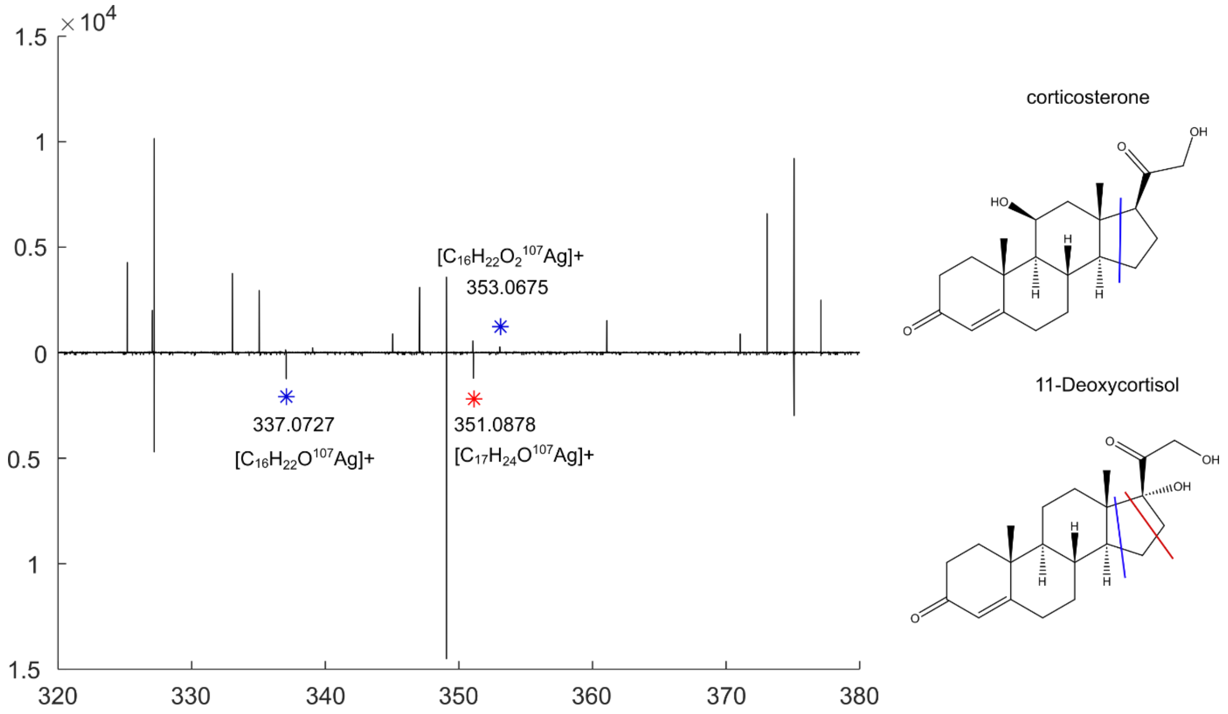

Figure S13. CID of the two regioisomers corticosterone and 11-deoxycortisol as $[M+Ag]^+$ induce c-c bond dissociation in the d-ring producing isomer specific fragment ions in MS^3 with less than 2 ppm mass difference compared to the theoretically predicted *m/z* values. Corticosterone produces a fragment ion at *m/z* = 353.0675 corresponding to d1 and 11-deoxycortisol produces a fragment ion at *m/z* = 337.0727 and 351.0878 corresponding to d1 and d2 respectively.

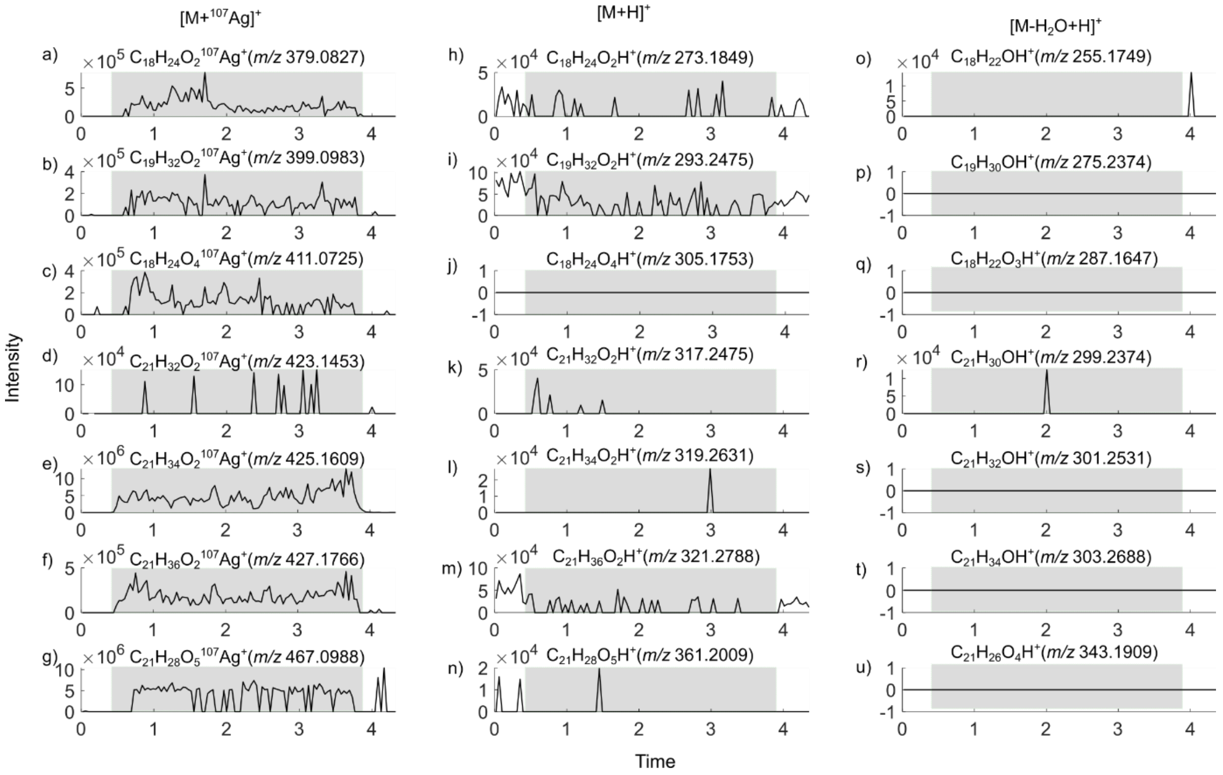

Figure S14 Extracted ion chronograms from a line scan of mouse brain tissue section: a-g) for *m/z* values corresponding to [M+^{107}Ag]$^+$, h-n) for *m/z* values corresponding to [M+H]$^+$, o-u) for *m/z* values corresponding to [M-H$_2$O+H]$^+$ steroid adducts from line scans of mouse brain tissue section. The liquid bridge is in contact with the tissue from time 0.69 to 3.84 and 0.49 min to 3.86 min for solvents with and without silver, respectively (marked in grey on each line scan). The intensity of silver adducts increases when the liquid bridge is in contact with the tissue.

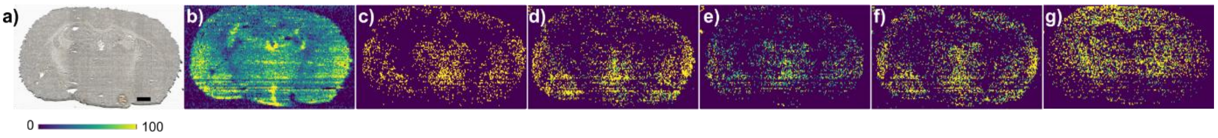

Figure S15 Ion images of putatively annotated Ag$^+$ cationized steroids in MS^1 show distinct distributions of neurosteroids/sterols in the mouse brain tissue a) optical image of the mouse brain section imaged. High-resolution ion images of *m/z* corresponding to silver cationized steroids b). pregnandiol *(m/z* 427.1766) c)**.** 11β-Hydroxyandrostenedione/7-ketodehydroepiandosterone *(m/z* 409.0933) d) tetrahydrodeoxycorticosterone (*m/z* 441.1559) e) cortisol/18hydroxycorticosterone (*m/z* 469.1144) f) tetrahydrocorticosterone *(m/z* 457.1508) h) androstenone/androstadienol (*m/z* 379.1191)

Schemes

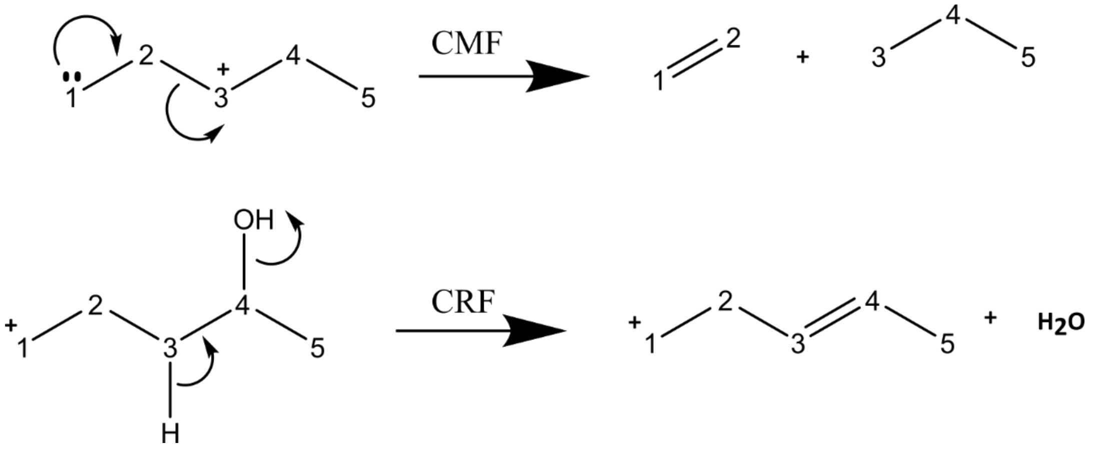

Scheme S1. Schematics of CRM and CRF. For CMF, number 1 represents an electronegative atom, while numbers 2 – 5 represent carbon atoms. For CRF, the numbers 1-5 represent carbon atoms.

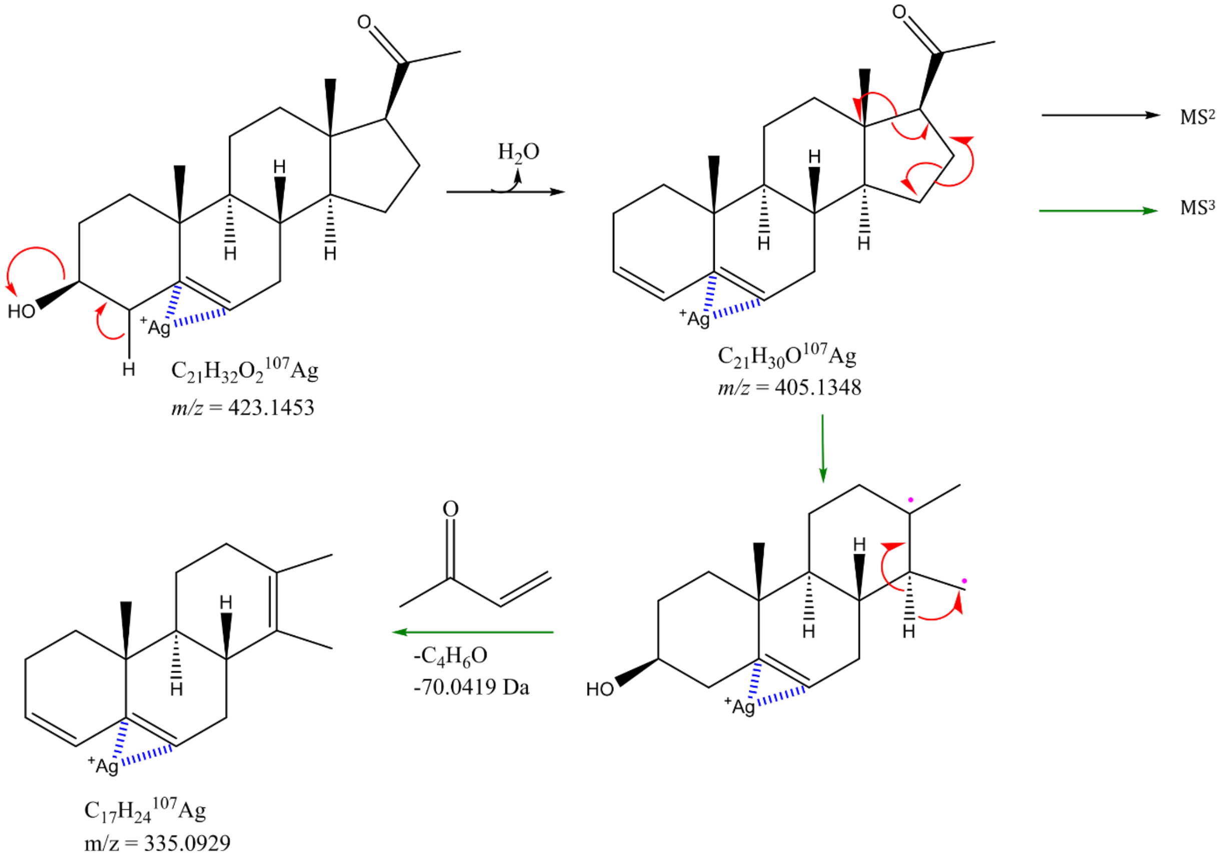

Scheme S2. A plausible fragmentation mechanism for d-ring fragmentation at d_2, which results in product ion at *m/z* 335.0929 observed in the MS^3 mass spectrum.

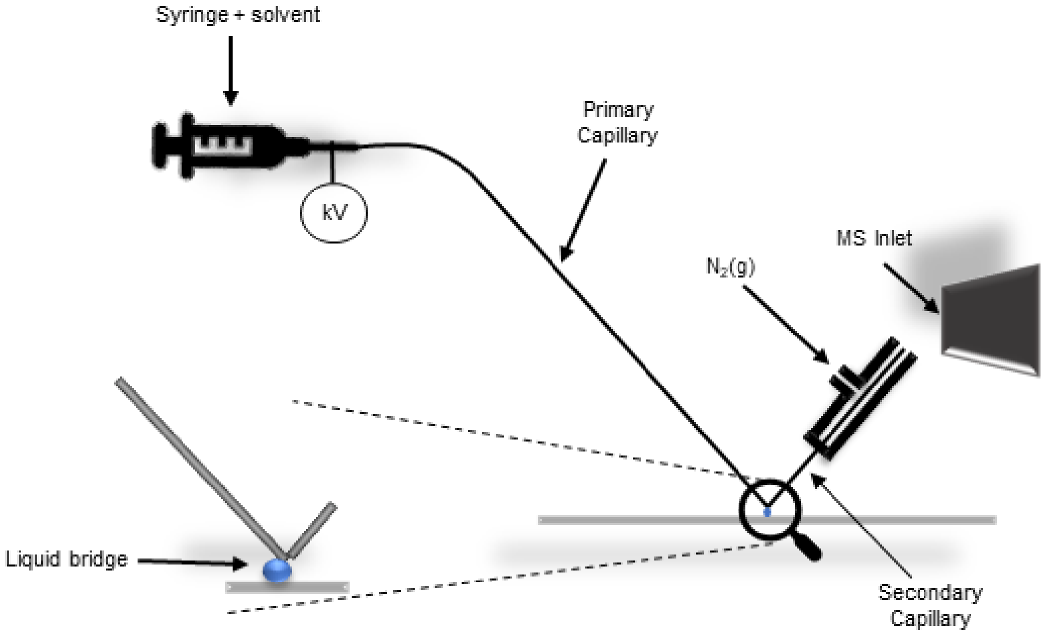

Scheme S3. Schematic representation of nano-DESI for the surface sampling of steroids from thin tissue sections.

Tables

Table S1: Sensitivity comparison between silver cautioned steroids and protonated adducts.

	$[M+^{107}Ag]^+$		M+H]$^+$	
steroids	Equation	R^2	Equation	R^2
Androstanedione	Y = 0.0307x + 0.0002	0.9982	Y = 0.0052x – 0.0004	0.9483
Androstanediol	Y = 0.0289x + 0.0003	0.9984	Y = 0.0075x + 0.0006	0.9447
Estradiol	Y = 0.0157x -0.0014	0.986	Y = 0.0001x + 0.00005	0.7131
estrone	Y = 0.0348x -0.0011	0.9368	Y = 0.0029x -0.0002	0.8498
Pregnanedione	Y = 0.229x + 0.0009	0.9699	Y = 0.0005x + 0.000006	0.9574

Table S2: Major *m/z* observed for 11-hydroxyprogesterone, 17-hydroxyprogesterone and 21hydroxyprogesterone as $[M+^{107}Ag]^+$

Steroid $[M+^{107}Ag]^+$	*m/z*	Fragment ion annotation	Accuracy (ppm)
11-hydroxyprogesterone	293.1895	$C_{21}H_{25}O$	-1.7
	311.2002	$C_{21}H_{27}O_2$	-1.6
	329.2105	$C_{21}H_{29}O_3$	-2.0
17-hydroxyprogesterone	296.1896	$C_{19}H_{25}O$	-1.3
	351.0867	$C_{17}H_{24}O^{107}Ag$	-1.5
	375.0867	$C_{19}H_{24}O^{107}Ag$	-2.0
21-hydroxyprogesterone	391.1178	$C_{20}H_{28}O^{107}Ag$	-0.8
